# The prognostic significance of heparanase expression in metastatic melanoma

**DOI:** 10.18632/oncotarget.12492

**Published:** 2016-10-06

**Authors:** Olga Vornicova, Ilanit Boyango, Sari Feld, Inna Naroditsky, Olga Kazarin, Yaniv Zohar, Yariv Tiram, Neta Ilan, Ofer Ben-Izhak, Israel Vlodavsky, Gil Bar-Sela

**Affiliations:** ^1^ Division of Oncology, Rambam Health Care Campus, Haifa, Israel; ^2^ Cancer and Vascular Biology Research Center, Bruce Rappaport Faculty of Medicine, Technion-Israel, Institute of Technology, Haifa, Israel; ^3^ Department of Pathology, Rambam Health Care Campus, Haifa, Israel

**Keywords:** heparanase, heparanase 2, melanoma, metastasis, survival

## Abstract

**Background:**

Heparanase expression is induced in many types of cancers, including melanoma, and promotes tumor growth, angiogenesis and metastasis. However, there is insufficient data regarding heparanase expression in the metastatic lesions that are the prime target for anti-cancer therapeutics. To that end, we examined heparanase expression in metastatic melanoma and its correlation with clinical parameters.

**Results:**

Heparanase staining was detected in 88% of the samples, and was strong in 46%. For the entire cohort of metastatic melanoma patients, no apparent correlation was found between heparanase staining intensity and survival. However, in a sub group of 46 patients diagnosed as stage IVc melanoma, strong heparanase staining was associated with reduced survival rates [hazard ratio=2.1; 95%CI 1.1-4.1, *p*=0.025].

**Material and Methods:**

Paraffin sections from 69 metastatic melanomas were subjected to immunohistochemical analysis, applying anti-heparanase antibody. The clinical and pathological data, together with heparanase staining intensity, were evaluated in a logistic regression model for site of metastasis and survival. Slides were also stained for the heparanase-homolog, heparanase-2 (Hpa2).

**Conclusion:**

Heparanase is highly expressed in metastatic melanoma and predicts poor survival of stage IVc melanoma patients, justifying the development and implementation of heparanase inhibitors as anti-cancer therapeutics.

## INTRODUCTION

Heparanase is the only mammalian endoglycosidase capable of cleaving heparan sulfate (HS) side chains of proteoglycans. This activity is responsible for remodeling of the extracellular matrix (ECM) underlying epithelial and endothelial cells and is highly implicated in cellular invasion associated with angiogenesis, inflammation and metastasis [1, 2]. Heparanase expression is increased in many types of tumors and this elevation is frequently associated with more aggressive disease and poor prognosis, most often due to increased tumor metastasis [3–5]. As such, heparanase is considered an attractive target for the development of anti-cancer drugs, and heparanase inhibitors are currently being evaluated in phase I/II clinical trials [6, 7]. Likewise, high levels of heparanase in melanoma patients were associated with poor prognosis [8, 9]. In pre-clinical studies, heparanase gene silencing in melanoma cells resulted in smaller tumors and decreased lung metastasis [10–12], associating with reduced cellular invasion, migration and adhesion [11–14]. Furthermore, heparanase gene silencing was associated with reduced Erk phosphorylation and cytokine expression [11], thought to play an essential role in melanoma progression [15, 16]. Importantly, while induction of heparanase was observed in all major types of cancer (i.e., carcinomas, sarcomas, hematological malignancies) [3, 4, 17, 18], systematic examination of its expression in the resulting metastases was evaluated in only small number of patients [19], without alluding to its clinical significance. This is critically important because metastases, rather than the primary tumor, are the prime target of anti-cancer therapeutics.

The aim of the current study was to examine the expression and significance of heparanase in metastatic melanoma. Utilizing immunohistochemistry, we found that most (88%) melanoma metastases stained positive for heparanase. Importantly, in stage IVc melanoma patients, high levels of heparanase were associated with poor prognosis (p=0.02). This result critically implies that heparanase not only enhances tumor cells dissemination but also promotes the growth and aggressiveness of the resulting metastases.

## RESULTS

While heparanase is reported to be induced in melanoma and to correlate with poor prognosis [8, 9], a systematic evaluation of its expression in metastatic lesions has not been sufficiently investigated. In order to examine heparanase levels in metastatic melanoma, 69 pathological samples from patients with melanoma metastases were subjected to immunostaining applying anti-heparanase antibody. These included metastasis to lymph nodes (n=15), lung (n=9), brain (n=21), skin (14), and other organs (n=10) (Table 2). Mean age of patients (43 males and 26 females) was 63.3 years (range, 35.8-88.1 years); 23 patients were diagnosed as stage IVa or IVb disease and 46 patients as stage IVc disease (patients with visceral metastasis other than lung and patients with metastatic disease to any site with elevated LDH levels). Demographic and clinical characteristics of the patients enrolled in this study are presented in Table 1.

**Table 1:** Patients characteristics

Number of patients	69
Sex (male\female)	43/26
Multiple vs single metastatic site	45/24
Patients with brain metastasis	25
Elevated LDH at diagnosis	22
Stage IVa+b	23
Stage IVc	46

**Table 2:** Heparanase staining in metastatic melanoma

Site of metastases	No of patients (%)	Negative or weak (0/+1)	Strong (+2)
Skin	14 (20)	5 (36)	9 (64)
Brain	21 (30)	12 (57)	9 (43)
Lung	9 (13)	7 (78)	2 (22)
Lymph nodes	15 (22)	9 (53)	7 (47)
Other	10 (14)	5 (50)	5 (50)
Total	69	37	32

**Figure 1:**
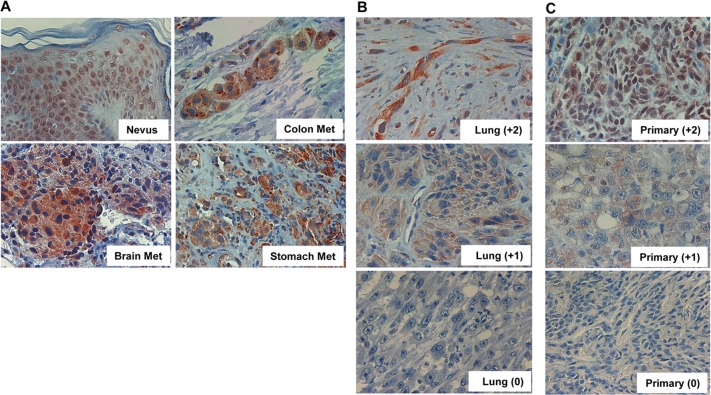
Heparanase expression in metastatic melanoma **A**. Melanoma metastases were subjected to immunostaining applying anti-heparanase antibody as described under ‘Materials and Methods’. Shown is representative staining of heparanase in brain, colon, and stomach melanoma metastases. Heparanase immunostaining in non-malignant nevi is also included as a reference for non-malignant lesion. Note nuclear localization of heparanase in non-malignant skin tissue, compared with diffused cytoplasmic distribution in melanoma metastases. Representative lung metastases exhibiting strong (+2), weak (+1) or no staining (0) of heparanase are shown in (**B**). Immunostaining of heparanase in non-matched primary melanomas exhibiting strong (+2), weak (+1) or no (0) staining is shown in (**C**). Original magnifications: x100.

Of the 69 specimens, 60 (88%) were stained positive for heparanase (Figure 1A); Lung metastases exhibiting strong (+2), weak (+1) or no staining (0) for heparanase are shown in figure 1B. Notably, heparanase staining appeared mostly diffused in the cytoplasm in the metastatic lesions compared with nuclear staining in non-malignant nevi (Figure 1A, Nevus; p<0.04). Although the primary lesions of the same patients were not available to us, staining of un-matched primary melanoma sections for heparanase showed comparable staining intensities (Figure 1C). Noteworthy, some nuclear staining of heparanase is retained in primary melanomas (Figure 1C, upper panels) but is not detected in metastatic melanomas (Figure 1A, 1B). Whereas staining intensity varied among patients (i.e., Figure 1B), the extent of staining (i.e., percent of cells stained positive) was typically high in all specimens that were stained positive. Clinical correlations were therefore analyzed in relation to heparanase staining intensity.

At the data cut-off, 57 patients were dead and 12 were alive. The median follow-up was 21.4 months (range, 0.5-128.6 months) for the entire group, 14.5 months (range, 0.5-110.5 months) for patients who died, and 53.7 months (range, 21.5-128.6 months) for those who were alive.

**Figure 2:**
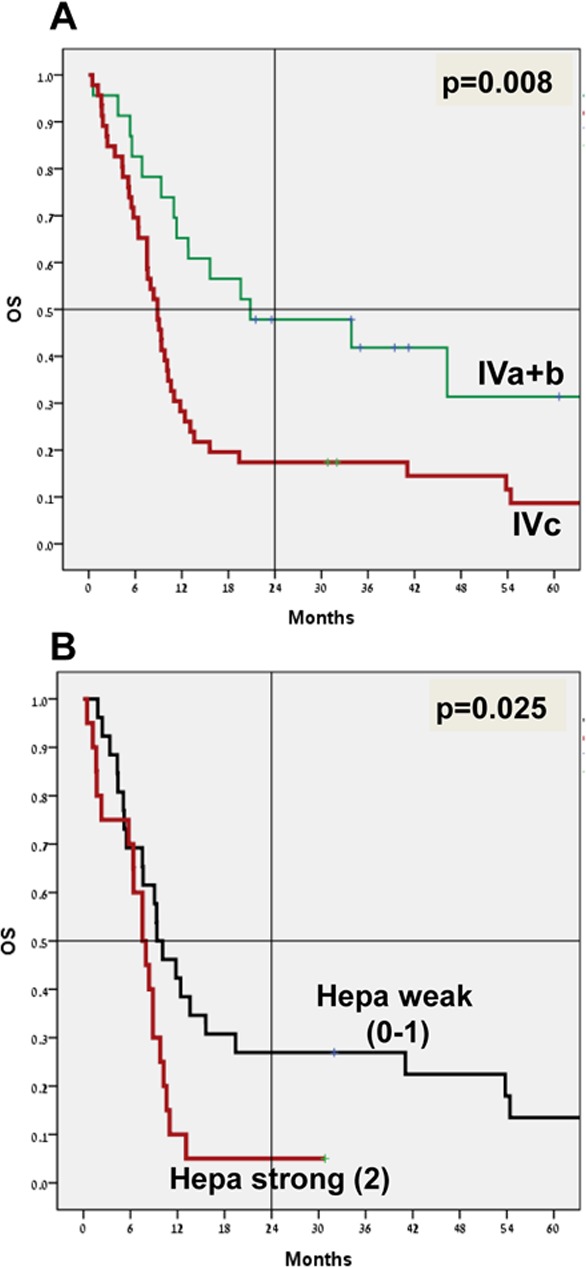
Patients' survival Overall survival analysis (OS; Kaplan-Meier) of stage IVa+b (n=23) vs. stage IVc (n=46) is shown in (**A**) (p=0.008). OS of stage IVc patients exhibiting weak (0/+1; n=26) vs. strong (+2; n=20) staining intensity of heparanase is shown in (**B**) (p=0.025). Note decreased survival rates of patients exhibiting strong staining of heparanase.

The main factors influencing survival in this patient cohort were stage at initial diagnosis (I-III vs. IV, p=0.009, hazard ratio (HR) 3.3), stage IVc vs. IVa and IVb, with 4-year survival of 15.2% vs. 39.1% (p=0.008, HR 2.2, 95%CI 1.2-4.1) (Figure 2A), and the presence of liver metastasis (4-year survival 0% vs. 29.6%; p=0.002, HR 2.7, 95%CI 1.5-5.1).

No apparent correlation was found between heparanase staining intensity and survival in the entire patient cohort. However, in a subgroup of patients with stage IVc disease (46/69 patients; Table 3), those who exhibited strong staining of heparanase (n=20) were endowed with poorer survival compared with stage IVc patients with weak or no staining of heparanase (n=26) (Figure 2B). The 4-year survival was 5% vs. 23.1%, with a median survival of 7.5 months vs. 9.4 months (p=0.025 for 4-year survival; HR 2.1, 95%CI 1.1-4.1) (Figure 2B).

**Table 3:** Clinical characteristics of stage IVc melanoma patients (46)

Male\female	29	17	
Stage at presentation	IV	13	
	III	9	
	I-II	15	
	NA	9	
Performance status (PS) at presentation	0	14	
	1	14	
	2	7	
	3-4	9	
	NA	2	
Number of metastatic sites at presentation	1	12 (8 brain mets)	
	2	16	
	3	18	
LDH at presentation	Normal	16	
	2*ULN	14	
	3*ULN and higher	10	
	NA	6	
Heparanase intensity	Negative (0)	Weak (+1)	Strong (+2)
Number of patients	7	21	18

**Figure 3:**
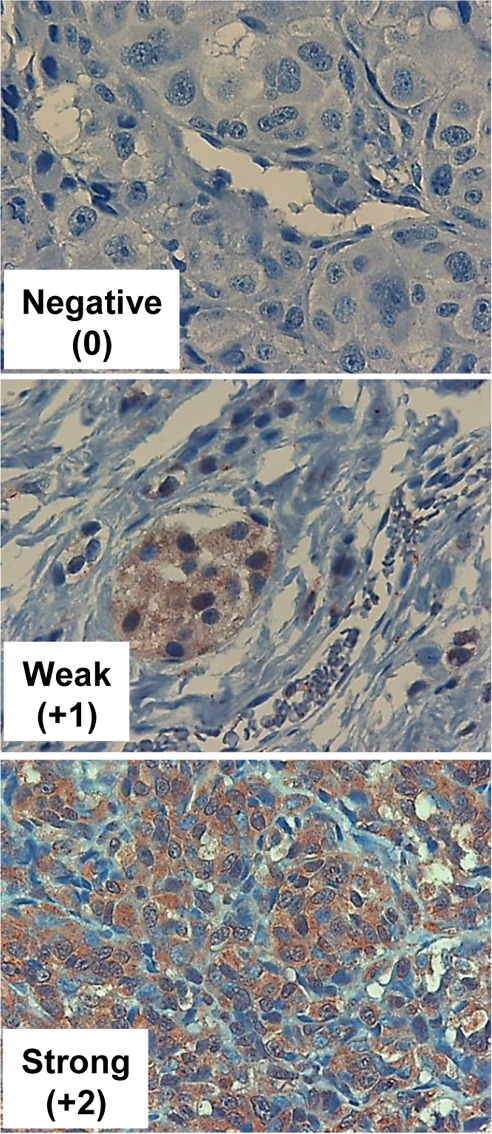
Hpa2 immunostaining Melanoma metastases were subjected to immunostaining applying anti-Hpa2 polyclonal antibody. Shown are representative photomicrographs of tumors that exhibit no (0, upper panel), weak (+1, middle panels) or strong (+2, lower panels) staining of Hpa2. Original magnification x100.

Metastatic melanoma specimens were also stained for the heparanase homolog, Hpa2 (Table 4). Hpa2 staining was detected in 68% of the cases, and 28% exhibited strong staining (Figure 3, lower panel). According to the metastatic site, Hpa2 staining was exceptionally low in brain metastasis. Thus, of the 18 cases of melanoma brain metastases, only one (6%) exhibited strong staining for Hpa2, significantly lower than the other sites of metastases (p=0.033; OR 0.1) (Table 4).

**Table 4:** Hpa2 staining in metastatic melanoma

Site of metastasis	No of patients (%)	Negative or weak (0/+1)	Strong (+2)
Skin	6 (13)	3 (50)	3 (50)
Brain	18 (38)	17 (94)	1 (6)
Lung	7 (15)	6 (86)	1 (14)
Lymph nodes	9 (19)	6 (67)	3 (33)
Other	7 (15)	2 (29)	5 (71)
Total	47 (100)	34 (72)	13 (28)

## DISCUSSION

Despite advances in treatment and surveillance, melanoma continues to claim approximately 9000 lives in the US annually (SEER 2013). Patients with stage IV melanoma are endowed with poor prognosis, with a mean survival of 8–10 months in large cohort analysis studies [20]. For a more accurate prognosis, patients with stage IV disease can be further subdivided into those with only cutaneous metastases (IVa), lung metastases (IVb), or other visceral metastases (IVc), to yield associated 5-year survival rates of 18.8%, 6.7%, and 9.5%, respectively [20]. Taking into consideration new treatment strategies that have become available during the past decade, including targeted therapy and immunotherapy, survival of stage IV melanoma patients has improved, with over 70% survival rates at one year with BRAF plus MEK inhibitor treatment or with PD-1 blockade, but long follow-up is needed to determine more accurate conclusions [21]. Due to this initial success, inhibitors targeting different pathways are currently being evaluated for anti-melanoma capacity, alone and in combination. Accordingly, heparanase inhibitors are presently evaluated clinically in melanoma patients among other indications [6, 7, 22]. This is based on compelling evidence that tie heparanase with tumor initiation, progression, metastasis, and chemo-resistance [4, 5, 23–28]. However, while the presence of heparanase at high levels has been documented in many types of cancers [4, 5, 7, 18], its abundance in the resulting metastases has not been sufficiently resolved. Here, we show that heparanase is readily detected in most (88%) melanoma metastases, regardless of their anatomical site (Table 2). Notably, in stage IVc melanoma patients, high levels of heparanase correlated with shorter survival rates (Figure 2B). This confirms and significantly expands previous report [19] and suggests that heparanase not only enhances the dissemination of tumor cells but also promotes the growth/aggressiveness of established lesions.

Unlike most epithelial cells, heparanase is expressed at relatively high levels by normal skin tissue [29]. In normal epidermal cells as well as non-malignant nevi, heparanase was localized primarily to the cell nucleus [9, 30] (Figure 1A, nevus). In striking contrast, heparanase appeared primarily diffused in the cytoplasm of metastatic melanoma cells (Figure 1A, 1B), in agreement with the notion that nuclear heparanase is associated with maintained cellular differentiation [31] and favorable outcome of cancer patients [31–33], whereas cytoplasmic heparanase represents a more secretable enzyme that promotes tumor progression.

Hpa2 was identified as a close homolog of heparanase based on sequence similarity [34], but its function in tumorigenesis is far less investigated. Like heparanase, Hpa2 binds heparin/HS with high affinity but lacks HS-degrading activity [35], the hallmark of heparanase. In fact, Hpa2 exhibits even higher affinity to HS than heparanase, suggesting that Hpa2 may inhibit heparanase activity by competing for the HS substrate [35]. Previously, we reported that Hpa2 expression was markedly elevated in head and neck carcinoma compared with normal epithelium, correlating with prolonged time to disease recurrence (follow-up to failure) and inversely correlating with tumor cell dissemination to regional lymph nodes [35], suggesting that Hpa2 functions as a tumor suppressor. Indeed, Hpa2 over-expression in head and neck cancer cells markedly reduces tumor growth [36]. Restrained tumor growth was associated with a prominent decrease in tumor vascularity (blood and lymph vessels), likely due to reduced Id1 expression, a transcription factor highly implicated in VEGF-A and VEGF-C gene regulation [36]. More recently, we found that Hpa2 is expressed at high levels in normal bladder transitional epithelium, whereas its expression is markedly decreased in bladder carcinoma [37]. Notably, bladder tumors that retain high levels of Hpa2 exhibit a higher degree of cell differentiation (low grade) and are less invasive (low stage), suggesting that Hpa2 functions as a tumor suppressor also in bladder cancer [37]. Significantly, only one of 18 cases of melanoma brain metastases exhibited strong staining of Hpa2 (Table 4), implying that Hpa2 possibly exhibits tumor suppressor properties for melanoma growth in the brain microenvironment.

Taken together, we describe for the first time a systematic examination of heparanase expression and clinical significance in melanoma metastases. Heparanase was readily detected in most metastases, and in stage IVc patients was associated with poor survival rates, suggesting that heparanase plays an important role in the progression of established metastatic lesions and, thus, is a promising target for the development of anti-cancer drugs. Studies examining the expression of heparanase in pairs of primary and resulting metastases of other types of cancer are currently ongoing.

## MATERIALS AND METHODS

### Materials

Paraffin blocks were obtained from 69 patients diagnosed with metastatic melanoma between the years 2007-2014 for whom follow-up records were available. Patients received standard of care treatment for metastatic melanoma, according to the time of diagnosis, including chemotherapy (dacarbazine, temodal, taxol), targeted therapy (anti-BRAF) and immunotherapy (anti-CTLA4 and anti-PD1), and were under the surveillance of the Division of Oncology at Rambam Health Care Campus in Haifa, Israel. Their performance was analyzed in correlation with pathological, demographic and clinical characteristics, including stage of disease (TNM) at the time of diagnosis, appearance of outlying metastases, and survival. Patients were excluded from the final analysis if only partial data were available from the medical record, or if it was impossible to prepare enough slides from the pathological material. It should be noted that the primary melanoma lesions were diagnosed and removed elsewhere, most often by a community dermatologist/surgeon, and were not available to us. The study was approved by the hospital's Helsinki Committee.

### Melanoma tissue array

We utilized melanoma tissue array (ME1004c; US Biomax, Rockville MD) to examine heparanase levels in non-malignant nevi and primary melanomas. The array included 100 human samples having stage and TNM characterization. Of these, 62 were diagnosed with malignant melanoma, 20 metastatic melanoma, and 18 nevi specimens.

### Immunohistochemistry

Biopsies were subjected to immunostaining, applying anti-heparanase and anti-heparanase-2 (Hpa2) antibodies essentially as described [36–38]. Briefly, slides were deparaffinized, rehydrated, and subjected to antigen retrieval by boiling (20 min) in 10 mM citrate buffer, pH 6.0. Following washes with phosphate buffered saline (PBS), slides were incubated with 10% normal goat serum in PBS for 60 min to block non-specific binding and incubated (20 h, 4°C) with rabbit anti-heparanase (#733) or anti-Hpa2 (#58) antibodies [36–38], diluted 1:100 in blocking solution. Slides were extensively washed with PBS containing 0.01% Triton X-100 and incubated with a secondary reagent (Envision) according to manufacturer's (Dako, Glostrup, Denmark) instructions. Following additional washes, color was developed with AEC reagent (Dako), and sections were counterstained with hematoxylin and mounted as described [36–38]. Immunostained specimens were examined independently by three senior pathologists who were blind to the clinical data of the patients. Heparanase staining in the melanoma cells was scored according to the cellular localization (nuclear vs. cytoplasmic), the extent (i.e., percent of positively-stained cells), and the intensity (0: none; +1: weak-moderate; +2: strong) of staining in the malignant cells. Specimens that were similarly stained with normal rabbit serum or by applying the above procedure but lacking the primary antibody yielded no detectable staining.

### Statistics

A comparison was made between the demographic data, the disease characteristics and the intensity of heparanase/Hpa2 staining by using a bivariate logistic regression. Cox regression model was used to determine factors influencing survival, illustrated by Kaplan-Meier curves. The level of significance selected to examine the various parameters in this study was set at *p*≤0.05. The data was processed using SPSS statistical software, version 21.0 (Chicago IL).
